# Robot-assisted radical surgery for colorectal cancer: technical advances, lymphadenectomy, natural orifice specimen extraction, and ongoing controversies

**DOI:** 10.3389/fsurg.2026.1951518

**Published:** 2026-10-08

**Authors:** Wanling Liu, Zhiqiang Ma, Jiangping Xie, Xin Wu, Qais Ahmad Baheen, Shaolu Zhang, Zheng Liu

**Affiliations:** Gastrointestinal Oncology Center, Arion Cancer Center (Beijing), Beijing, China

**Keywords:** colon cancer, evidence appraisal, lymphadenectomy, natural orifice specimen extraction surgery, oncological outcomes, rectal cancer, robot-assisted surgery

## Abstract

Robot-assisted surgery expands the technical options for colorectal cancer resection, but its clinical value depends on the operative setting and the outcome assessed. This narrative review examines lymphadenectomy and natural orifice specimen extraction surgery (NOSES), distinguishing colon from rectal cancer and technical feasibility from demonstrated clinical benefit. Literature published through 1 July 2026 was considered. Randomized evidence in rectal cancer is heterogeneous: ROLARR and COLRAR did not demonstrate superiority for their respective primary outcomes, whereas REAL reported better selected perioperative outcomes and lower 3-year locoregional recurrence after robotic surgery. These findings do not establish an overall survival advantage or a benefit mediated specifically by more extensive lymphadenectomy. Evidence for robotic colectomy remains less definitive. Lymph node yield, dissection extent, vascular ligation and specimen quality should be evaluated separately, with attention to geographical differences in practice. Robotic NOSES may reduce pain and wound morbidity in selected patients; a recent randomized trial supports short-term benefits, while long-term oncological equivalence remains unproven. Selection bias, limited external validity, cost and variable surgeon experience constrain broader conclusions. Adoption should therefore be guided by procedure-specific evidence, appropriate oncological resection and patient priorities rather than technical capability alone.

## 1 Introduction

Surgical resection remains central to the curative treatment of resectable colon and rectal cancers. Its objectives include negative margins, appropriate regional lymph node assessment and preservation of function. Although these objectives are shared, the operations are not interchangeable: colectomy addresses mesocolic anatomy and regional vascular drainage, whereas rectal resection requires particular attention to the mesorectal plane, circumferential resection margin and pelvic autonomic nerves. The indications for additional lateral pelvic lymph node dissection also belong to the rectal cancer setting (1, 2).

Robot-assisted surgery offers magnified three-dimensional visualization, articulated instruments and stable instrument control. These features provide a plausible technical rationale for difficult pelvic dissection and intracorporeal reconstruction. Their presence, however, does not demonstrate that a particular operation improves recovery, cancer control or survival. Comparative trials have produced different results across endpoints and surgical settings, making it necessary to distinguish what a robotic system enables from what clinical studies have established (3–6).

Natural orifice specimen extraction surgery (NOSES) addresses a separate component of minimally invasive surgery: the route by which the resected specimen leaves the body. It avoids an additional abdominal extraction incision by using a transanal or transvaginal route, while abdominal access ports may still be required. NOSES can accompany either laparoscopic or robotic resection. Consequently, an outcome associated with natural-orifice extraction should not automatically be attributed to the robotic platform (7).

This review focuses on the evidence for robot-assisted lymphadenectomy and NOSES. It considers colon and rectal cancer separately, evaluates the methodological strength and applicability of comparative studies, and identifies the questions that remain unresolved. Recovery, costs, training and emerging technologies are discussed insofar as they affect these operative decisions.

## 2 Methods and search strategy

The initial literature searches were conducted in PubMed, Web of Science, Scopus and Embase on 1 July 2026, without a lower publication-date limit. Studies published within the preceding 10 years were prioritized, alongside relevant earlier evidence. The review addressed robotic colon and rectal cancer surgery, lymphadenectomy and natural orifice specimen extraction (NOSES). Search terms covered robotic or robot-assisted surgery, colon or rectal cancer, colectomy, NOSES, complete mesocolic excision and lateral lymph node dissection. Targeted PubMed searches and reference checks were last completed on 9 September 2026, retaining 1 July 2026 as the publication cutoff.

English- and Chinese-language clinical reports were considered. Publications were selected for their relevance to the surgical population, comparison and outcome being discussed. Randomized trials and follow-up reports were prioritized for treatment effects, with comparative observational studies, systematic reviews and guidelines providing complementary evidence. Case reports and technical series informed feasibility only; trial protocols and reports from other specialties were not used to support colorectal efficacy claims. Evidence was appraised qualitatively according to study design, comparator, sample size, follow-up and endpoint relevance. Reports from the same trial were identified as related publications. Selection and synthesis were narrative, without formal risk-of-bias scoring or quantitative pooling.

## 3 Technical evolution and procedure-specific clinical evidence

### 3.1 Technical capabilities and outcome definitions

The transition from open surgery to minimally invasive approaches has expanded the available routes to an oncologically appropriate resection. Robotic platforms add articulated instruments, motion scaling, tremor filtration and console-controlled visualization to this repertoire. These capabilities can facilitate exposure and instrument movement in confined spaces. In this review, technical precision refers to anatomical identification, controlled dissection or suturing, rather than an assumed reduction in complications or improvement in survival.

Evidence should follow the same distinction. Operative time and conversion reflect aspects of technical performance; lymph node yield, margin status and specimen plane describe pathological quality; recurrence, survival and validated functional outcomes assess clinical consequences. Improvement in one domain cannot be presumed to extend to the others. Figure 1 summarizes the technical framework, and Table 1 compares the principal clinical studies.

**Figure 1:**
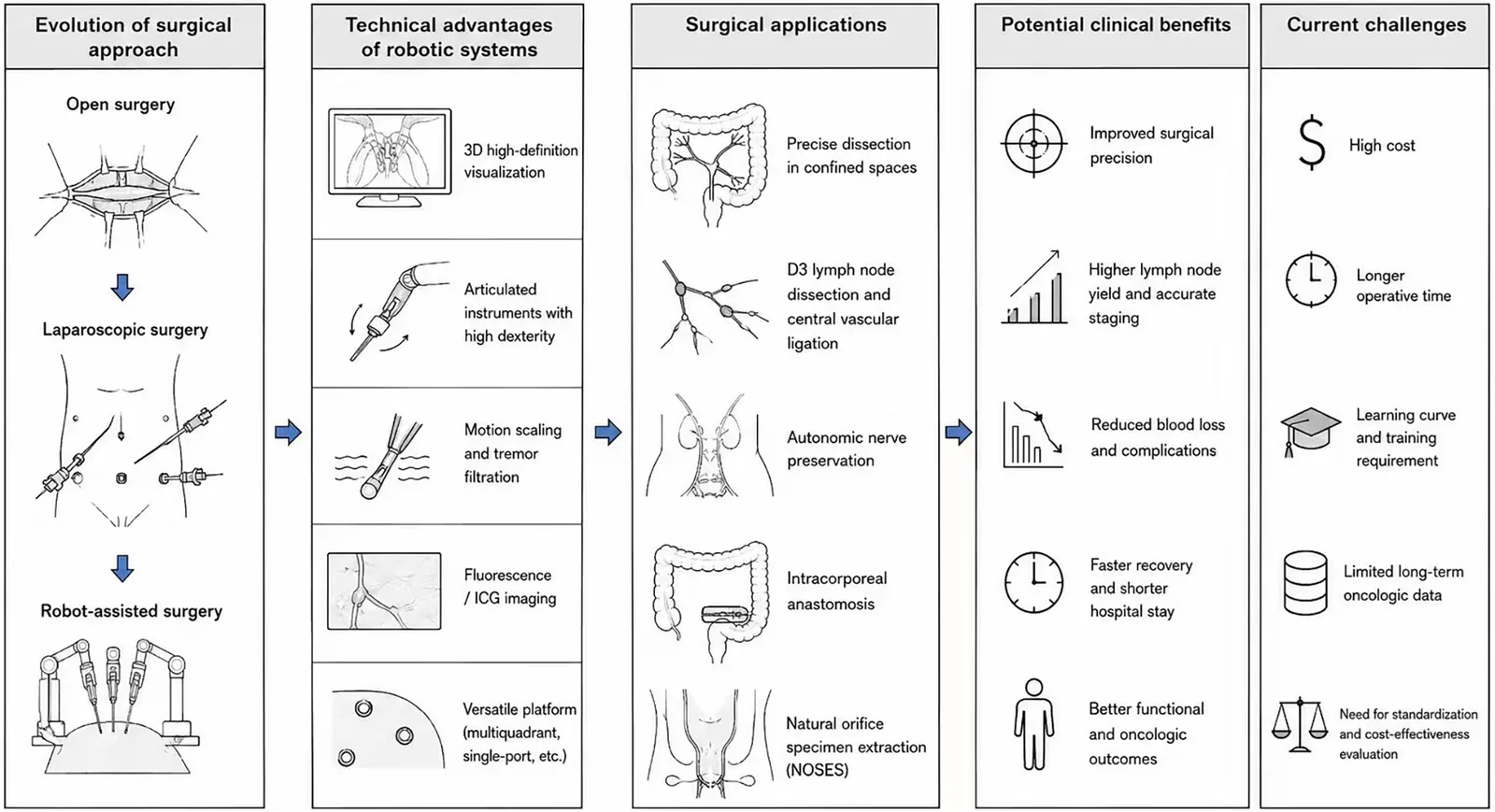
Technical framework of robot-assisted colorectal surgery. The schematic links technical capabilities to operative applications and potential clinical effects. These links are conceptual and do not establish universal benefit. Nodal yield is a pathological measure, and clinical or oncological effects vary by procedure and endpoint; the randomized evidence is summarized in Table 1. D3/CME-related colon dissection should be distinguished from rectal TME and selective lateral pelvic dissection. ICG lymphatic mapping, perfusion assessment and tumor localization are separate applications.

**Table 1:** Comparative clinical evidence for robotic colectomy and rectal resection.

Study and design	Population and comparison	Main finding	Limit and clinical interpretation
ROLARR, 2017 Multicenter randomized trial (3)	471 randomized; robotic versus laparoscopic rectal resection; 29 sites, 10 countries.	Conversion: 8.1% versus 12.2%; adjusted OR 0.61 (95% CI 0.31-1.21). Primary endpoint not significantly different.	Variable robotic experience; earlier platform era. A numerical difference does not establish superiority.
COLRAR, 2023 Multicenter randomized trial (4)	295 randomized; middle/low rectal cancer; robotic versus laparoscopic TME.	Complete TME: 80.7% versus 77.1%; no significant primary-outcome improvement.	Premature termination for poor accrual reduced precision. Positive subgroup findings do not replace the primary analysis.
REAL, 2022 and 2025 Two reports of one randomized trial (5, 6)	1,240 randomized; 1,171 in primary analysis; middle/low rectal cancer; 11 Chinese centers.	Lower margin positivity and grade II or higher complications in the short-term report. Lower 3-year locoregional recurrence in follow-up; no significant OS difference.	Post-randomization exclusions and setting affect applicability. Supports selected rectal-cancer benefits, not a colectomy effect or a mechanism mediated by wider lymphadenectomy.
Park et al., 2012 and 2019 Randomized right-colectomy cohort and follow-up (8, 9)	71 randomized; 70 in the initial analysis; robotic versus laparoscopic right colectomy.	No significant recovery or pathological advantage in the initial report. No significant 5-year DFS or OS difference; longer surgery and higher costs with robotics.	Small cohort and older technology. Insufficient to establish equivalence or exclude a clinically important contemporary benefit.
Lin et al., 2025 Retrospective propensity-matched study (10)	Right colectomy: 50 robotic versus 200 laparoscopic; left colectomy: 129 versus 258.	Recovery measures favored robotics. Right-sided node yield: 31.4 versus 26.8. Major complication rates were not significantly different.	Single-center nonrandomized comparison; operative and care-pathway confounding. Nodal yield is a pathological endpoint.
López-Huerta et al., 2025 Retrospective platform comparison (11)	284 rectal cancer patients; da Vinci S and Xi used in different calendar periods.	OS comparison: HR 0.54 (95% CI 0.27-1.07), *P* = 0.07; no significant local recurrence or DFS differences.	Temporal and clinical confounding. Does not establish that platform advancement improves survival.

Studies are grouped by operative population and design. Qualitative appraisal is not a formal GRADE rating. Reports from the same randomized cohort are not independent trials. TME, total mesorectal excision; OR, odds ratio; HR, hazard ratio; CI, confidence interval; DFS, disease-free survival; OS, overall survival.

### 3.2 Robotic colectomy

In colon cancer, robotic assistance is relevant to mesocolic dissection, vascular exposure and intracorporeal anastomosis. These technical tasks differ from dissection within the low pelvis. In an early randomized trial of right colectomy, 71 patients were randomized and 70 analyzed. Robotic and laparoscopic groups had similar hospital stay, complications and pathological findings, but robotic surgery took longer and incurred higher hospital costs. The later report from this small randomized cohort found no significant difference in 5-year disease-free or overall survival. Its size and older technology limit definitive conclusions about contemporary platforms, but it does not support a general survival advantage for robotic colectomy (8, 9).

More recent observational findings are favorable for selected recovery measures. Lin et al. analyzed right and left colectomy separately after propensity matching. For right colectomy, the robotic group had a higher mean lymph node yield than the laparoscopic group (31.4 versus 26.8; *P* = 0.028), whereas the corresponding left-sided comparison was not significant. Hospital stay and pain measures favored robotics, but major complication rates did not differ significantly. These associations remain susceptible to selection, perioperative care and operative-technique differences; a pathological yield advantage cannot establish better cancer control (10).

Accordingly, colectomy studies should specify side of resection, anastomotic technique, extraction incision, lymphadenectomy extent and enhanced recovery practice. Comparisons that change several of these factors alongside the platform cannot isolate the contribution of robotic assistance. Benefits observed in rectal cancer trials should not be applied directly to colectomy.

### 3.3 Robotic rectal resection

Rectal cancer provides a stronger anatomical rationale for robotic assistance because pelvic access, distal dissection and autonomic nerve preservation can be technically demanding. Nevertheless, randomized results have not been uniform. ROLARR randomized 471 patients across 29 sites in 10 countries. Conversion occurred in 8.1% of robotic and 12.2% of laparoscopic procedures, without a statistically significant difference (adjusted odds ratio 0.61, 95% confidence interval [CI] 0.31-1.21; *P* = 0.16). Participating surgeons had varying robotic experience. COLRAR, terminated early because of poor accrual, randomized 295 patients and did not demonstrate a significant improvement in complete total mesorectal excision (TME) (3, 4).

The larger REAL trial enrolled patients with middle or low rectal cancer at 11 Chinese centers. Of 1,240 randomized patients, 1,171 were included in the modified intention-to-treat analysis. The short-term report found lower circumferential margin positivity and fewer 30-day complications of Clavien-Dindo grade II or higher after robotic surgery. These were secondary outcomes within a trial designed around locoregional recurrence (5).

The subsequent REAL report provides relevant long-term evidence. At a median follow-up of 43 months, 3-year locoregional recurrence was 1.6% after robotic and 4.0% after laparoscopic surgery (hazard ratio [HR] 0.45, 95% CI 0.22-0.92). Three-year disease-free survival was also higher in the robotic group, while overall survival did not differ significantly. Thus, selected oncological benefits have been demonstrated in this trial population. Generalization requires attention to surgical expertise, patient eligibility, the single-country setting and post-randomization exclusions. The trial compared complete operative approaches and did not establish that lymph node yield or wider lymphadenectomy caused the recurrence benefit (6).

### 3.4 Comparisons between robotic platforms

Platform comparisons answer a different question from robotic versus laparoscopic trials. In the retrospective series by López-Huerta et al., the da Vinci S and Xi systems were used during different periods, from 2008 to 2018 and 2018-2021, respectively. Five-year overall survival was numerically higher with Xi (86.8% versus 64.9%), but the estimate was imprecise (HR 0.54, 95% CI 0.27-1.07; *P* = 0.07). Local recurrence and disease-free survival did not differ significantly. These results do not establish a survival advantage attributable to platform advancement. Calendar time, case selection, comorbidity and changes in perioperative or oncological treatment are potential confounders (11).

## 4 Advances and challenges in lymphadenectomy

### 4.1 Lymph node yield and oncological quality

Lymph node assessment contributes to pathological staging and treatment planning. The number examined is influenced by the anatomical resection, pathological processing, patient and tumor biology, and preoperative treatment. The commonly used benchmark of at least 12 examined nodes in colon cancer supports staging adequacy; it is not a dose-response target proving that every additional node improves survival. Neoadjuvant treatment further complicates comparisons of nodal counts in rectal cancer (1, 2).

Several concepts therefore require separate reporting. Lymph node yield is a count; lymphadenectomy extent describes the nodal stations removed; vascular ligation specifies where a vessel is divided; and specimen-plane assessment evaluates the integrity of the mesocolic or mesorectal envelope. Complete mesocolic excision (CME), D3 dissection and central vascular ligation are related components of some colectomy techniques, but they are not synonyms. TME describes a rectal resection plane, while lateral pelvic dissection addresses nodal compartments outside the mesorectum. Table 2 sets out these distinctions (1, 2, 12).

**Table 2:** Lymphadenectomy concepts, supporting evidence and unresolved questions.

Concept and setting	What is measured or decided	Evidence and limitation	Clinical implication
Lymph node yield Colon and rectal cancer	Number of nodes identified and examined pathologically.	Affected by surgical extent, pathology, biology and preoperative therapy. The colon-cancer staging benchmark is not a survival dose-response target. (1, 2)	Interpret with margin and specimen quality. More nodes alone do not prove better cancer control.
CME and D3 dissection Primarily colon cancer	CME concerns the mesocolic plane/envelope; D3 specifies more central nodal clearance. Vascular ligation is a related but separate technical choice.	Guideline approaches vary. RELARC did not demonstrate significant overall DFS superiority at 3 years or OS superiority at 5 years; subgroup findings require context. (1, 12, 14, 15)	Describe the actual nodal territory. Do not treat CME, D3 and central vascular ligation as interchangeable.
IMA ligation Rectal and left-sided surgery	Site of arterial division versus the extent of associated lymphatic dissection.	High tie is not universally required; low tie with appropriate lymphatic clearance is accepted in selected rectal practice. (2)	Separate the vascular decision from the nodal decision and consider perfusion and anastomotic reach.
Mesorectal excision Rectal cancer	Integrity of the resection plane, CRM and appropriate distal clearance.	Randomized trial results differ; REAL provides positive selected pathological and recurrence findings. (4–6)	Assess rectal-specific quality and function. These findings cannot establish a benefit for colon surgery.
Lateral pelvic dissection Selected rectal cancer	Clearance of lateral compartments outside the mesorectum.	Indications depend on height, imaging, neoadjuvant strategy and regional practice. Guidance differs across settings. (2, 12)	Stage III disease alone is not an indication for routine lateral clearance. Distinguish indication from platform choice.
ICG lymphatic mapping Selected colorectal surgery	Visualized drainage pathways and nodal retrieval.	A prospective propensity-matched study reported favorable associations during laparoscopic surgery. It does not establish a robotic effect or causal survival benefit. (16)	Separate mapping from perfusion assessment and tumor localization; fluorescence alone does not justify extending resection.

CME, complete mesocolic excision; IMA, inferior mesenteric artery; CRM, circumferential resection margin; ICG, indocyanine green; DFS, disease-free survival; OS, overall survival. Anatomical decisions should be assessed independently of the technical platform.

### 4.2 Colon cancer and the extent of mesocolic dissection

Statements about a universal standard of D3 dissection require geographical context. ASCRS guidance does not recommend routine extended lymphadenectomy for all colon cancers, whereas JSCCR guidance relates dissection extent to clinical nodal findings and depth of invasion. The relevant nodal territory and vascular anatomy should be stated explicitly when describing a robotic operation. Neither the platform nor a stage label alone determines the appropriate extent of resection (1, 12).

Observational studies have reported survival associations favoring D3 over D2 dissection, including a propensity-matched pT2 colorectal cancer cohort. Such data address lymphadenectomy extent rather than a robotic effect and remain vulnerable to residual confounding (13). Randomized evidence warrants a more restrained interpretation. RELARC compared CME with D2 dissection during laparoscopic right colectomy, with 995 patients in its primary analysis. The 3-year disease-free survival difference did not reach statistical significance (HR 0.74, 95% CI 0.54-1.02; *P* = 0.06) (14).

The 5-year RELARC report likewise found no significant overall survival or cancer-specific survival difference in the full analysis population. Favorable findings in selected pathological subgroups, including pN2 disease, merit further evaluation but do not establish routine benefit for every patient. Both reports concern laparoscopic dissection extent, not superiority of robotic assistance. They illustrate why a wider clearance cannot be assumed to improve outcomes merely because a platform makes it technically achievable (15).

### 4.3 Rectal cancer, vascular ligation and lateral pelvic nodes

For rectal cancer, appropriate mesorectal excision and margin clearance should be assessed alongside preservation of pelvic autonomic structures. High ligation of the inferior mesenteric artery is not a universal requirement. ASCRS guidance accepts routine low tie with associated lymphatic clearance in appropriate cases, reserving high tie for selected circumstances such as suspicious apical nodes or the need for additional length. The level of arterial division must be distinguished from removal of the relevant lymphatic tissue (2).

Lateral pelvic lymph node dissection is a separate decision, rather than an automatic extension of surgery for all stage III tumors. Selection depends on tumor height, lateral nodal findings on imaging, neoadjuvant treatment and regional practice. Japanese and Western strategies differ in their use of prophylactic and selective dissection. A review of robotic surgery should describe these contexts instead of presenting one approach as a globally accepted standard (2, 12).

The robot may facilitate access to lateral compartments and dissection around nerves and vessels. However, technical feasibility does not establish that robot-assisted lateral dissection improves survival, functional preservation or local control compared with an equally appropriate dissection performed by another approach. Studies should distinguish the indication for lateral clearance from the choice of platform and evaluate both oncological and urinary or sexual outcomes.

### 4.4 Fluorescence guidance and the limits of surrogate endpoints

Fluorescence-based tumor localization, perfusion assessment and lymphatic mapping address different questions. Evidence for one application should not be used to claim effectiveness in another. Bao et al. reported greater nodal retrieval and an association with survival in a prospective propensity-matched study of fluorescence-guided lymphatic mapping. The operations were laparoscopic, so these findings cannot demonstrate a robotic platform effect; nonrandomized allocation also limits causal interpretation (16).

Identification of additional fluorescent drainage pathways does not itself prove that extending resection along those pathways improves cancer outcomes. Nor does a higher yield identify the mechanism behind a survival association. Future comparative studies should document the anatomical dissection, specimen assessment, pathological processing and neoadjuvant treatment, and link these measures to recurrence and function. Until such links are demonstrated, improved exposure and nodal retrieval should be described as technical or pathological findings rather than established survival benefits.

## 5 Natural orifice specimen extraction surgery

### 5.1 Principles and operative workflow

NOSES combines an oncological resection with retrieval through a natural orifice. It should not be confused with natural orifice transluminal endoscopic surgery (NOTES), which describes a different access concept. Robotic NOSES generally retains abdominal ports; its intended reduction in abdominal wall trauma concerns the extraction incision, not elimination of all abdominal wounds. The resection margins and indicated lymphadenectomy should be determined independently of the intended retrieval route (7).

The operative sequence includes robotic mobilization and oncological dissection, specimen protection, transanal or transvaginal retrieval, and reconstruction. The order and method of reconstruction vary with the procedure. Articulated instruments may facilitate intracorporeal suturing, but the clinical effects of the robotic platform, anastomotic technique and extraction route should be evaluated separately. Figure 2 illustrates selection and workflow considerations; Table 3 summarizes the evidence and its limitations.

**Figure 2:**
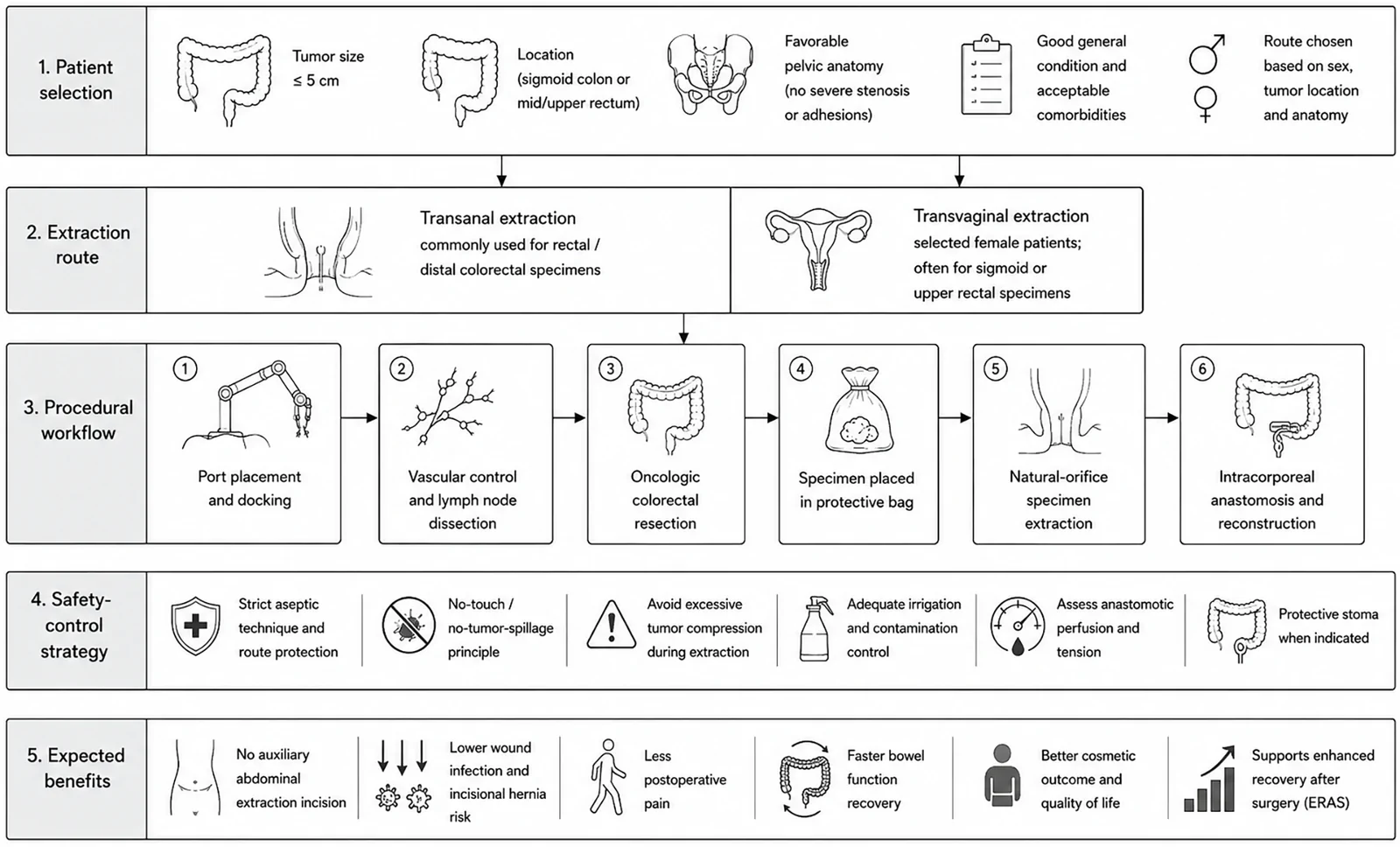
Selection and workflow for robotic NOSES. The schematic describes route selection, protected retrieval and reconstruction. The 5 cm size example reflects selection in some published cohorts and is not a universal eligibility cutoff; specimen bulk and shape, extraction route, anatomy and safe intact retrieval determine feasibility. “Expected benefits” are outcome-dependent potential benefits, not evidence of long-term oncological equivalence. Abdominal access ports may remain necessary, and the order of reconstruction varies by procedure. The evidence and its limitations are summarized in Table 3.

**Table 3:** Evidence for robotic NOSES and the boundaries of clinical inference.

Evidence or decision	Population and comparator	Supported finding	Unresolved issue and implication
Li et al., 2026 Randomized trial (21)	150 randomized; 140 analyzed; robotic NOSES versus conventional robotic TME in mid-rectal cancer.	Selected pain, recovery and wound outcomes favored NOSES; no significant overall complication or leakage difference.	Short-term report with post-randomization exclusions. Does not establish long-term oncological equivalence.
Liu et al., 2024 Multicenter retrospective matched study (20)	318 matched pairs from 8 centers; pathologically early-stage rectal cancer; robotic NOSES versus robotic abdominal extraction.	Fewer wound complications and favorable selected recovery and quality-of-life measures; overall complication rates not significantly different.	Experienced centers and restrictive pathological selection. Matching does not remove unmeasured selection bias.
Liu et al., 2020; Li et al., 2023 Matched observational cohorts (24, 25)	49 and 39 matched pairs, respectively; robotic natural-orifice versus abdominal extraction.	Lower pain or earlier recovery; no significant differences in reported 3-year survival endpoints.	Small samples and limited events. Nonsignificance cannot demonstrate equivalence or noninferiority.
Zhan et al., 2024 Meta-analysis (26)	6 observational studies, 717 patients; robotic natural-orifice versus abdominal extraction.	Pooled associations favored pain and wound outcomes. No significant difference in leakage, ileus or 3-year survival.	Inherits component-study confounding. Recovery estimates were heterogeneous; pooled observational evidence remains observational.
Feng et al., 2021; Ye et al., 2023 Retrospective comparisons (28, 29)	Robotic NOSES versus conventional laparoscopy; or robotic versus laparoscopic NOSES.	Favorable selected short-term outcomes in the robotic groups.	The first comparison changes platform and extraction route together; the second included only 46 patients. Neither isolates a robust long-term platform effect.
Specimen size and route (17–19)	Selection depends on specimen bulk and shape, tumor site, anatomy, baseline function and patient preference.	Approximately 5 cm was used in some cohorts; larger specimens were accepted selectively in another series. Route-specific functional findings were mixed.	Avoid a universal size cutoff or claim that one route is superior for all women. Report failed or abandoned NOSES attempts.
Protection and follow-up (7)	Intact oncological resection, protected extraction and appropriate reconstruction.	Consensus supports route protection, avoidance of tumor disruption and attention to contamination.	Procedural safeguards are not proof of oncological safety. Follow recurrence, hernia, anal and sexual function over time.

NOSES, natural orifice specimen extraction surgery; TME, total mesorectal excision. The ability to obtain a negative margin or an adequate nodal count is not equivalent to demonstrated long-term oncological noninferiority. Narrative descriptions of study design are not formal risk-of-bias ratings.

### 5.2 Patient selection and generalizability

Tumor location, specimen bulk, mesenteric thickness, pelvic anatomy, extraction-route suitability and surgeon experience influence whether NOSES can be completed without excessive compression or tissue injury. A diameter of approximately 5 cm has been used in selected series, but it is not a universal eligibility threshold. One 162-patient robotic series allowed larger specimens when their shape permitted longitudinal extraction; another transvaginal series was limited to selected sigmoid or upper rectal cancers smaller than 5 cm. These differing criteria underscore the need to describe the actual specimen and route rather than impose a single tumor-size rule (17, 18).

Transanal and transvaginal extraction have different anatomical and functional implications. A retrospective comparison in women undergoing robotic mid-rectal resection reported some anal-function advantages with transvaginal extraction, while sexual-function findings varied by subgroup. Route selection should therefore consider baseline function, anatomy and informed patient preference, without assuming that one route is uniformly preferable (19).

Published cohorts often exclude emergencies, extensive adhesions, unfavorable extraction anatomy or tumors requiring complex multivisceral resection. Such exclusions describe the populations studied; they should not automatically be presented as universal contraindications. More importantly, successful results in selected patients at experienced centers do not establish comparable outcomes in unselected practice. Propensity matching balances measured variables but cannot fully account for unrecorded extraction difficulty, surgeon judgment or technical experience. If safe intact retrieval is doubtful, an abdominal extraction incision remains an appropriate option rather than a failure of oncological surgery (7, 17, 20).

### 5.3 Comparative evidence and oncological interpretation

The evidence base includes observational comparisons, meta-analyses of those comparisons and emerging randomized data. A 2026 trial randomized 150 patients with mid-rectal cancer and analyzed 140 after exclusions. It reported lower pain scores, earlier recovery milestones and fewer wound complications with robotic NOSES, while overall complication rates, anastomotic leakage and pathological measures did not differ significantly. These findings strengthen the evidence for selected short-term benefits. They do not establish long-term cancer safety or equivalence, and the modest sample and post-randomization exclusions should remain visible when interpreting the results (21).

A multicenter retrospective study from eight high-volume Chinese centers compared robotic NOSES with robotic transabdominal extraction in pathologically early-stage rectal cancer. Among 318 matched pairs, wound complications and several recovery measures favored NOSES, whereas overall complication rates did not differ significantly. The multicenter design improves the breadth of experience represented, but restriction to early pathological stages and experienced surgeons limits applicability to more advanced disease and routine adoption (20).

Smaller studies provide complementary information. Ye et al. compared 33 robotic NOSES cases with 50 robotic transabdominal extractions in a retrospective series; Tao et al. reported a retrospective comparison of the I-type F technique for lower rectal cancer. Neither report is a randomized trial. Their short-term findings should be interpreted as selected institutional experience (22, 23).

Longer follow-up has been reported in matched cohorts. Liu et al. studied 49 matched pairs undergoing robotic rectal resection, and Li et al. studied 39 matched pairs undergoing robotic sigmoid or rectal resection. Both reported no significant between-group survival differences. Small samples and few events, however, leave uncertainty about clinically meaningful differences in recurrence or survival; absence of statistical significance is not proof of equivalence or noninferiority (24, 25).

A meta-analysis of six observational studies involving 717 patients associated robotic NOSES with less pain and fewer wound infections, but did not find significant differences in anastomotic leakage, postoperative ileus or 3-year survival. Its conclusions inherit the selection and confounding limitations of the component cohorts. The related review of nine retrospective studies should likewise be read as a synthesis of nonrandomized evidence, rather than as independent confirmation of oncological equivalence (26, 27).

Comparator choice is crucial. A comparison of robotic NOSES with conventional laparoscopic resection changes both the platform and extraction route, as in the propensity-matched study by Feng et al. Such a design cannot isolate the contribution of NOSES. Conversely, the small retrospective comparison of robotic and laparoscopic NOSES addresses platform choice while retaining natural-orifice extraction, but its sample of 46 patients limits inference. Reviews pooling predominantly laparoscopic NOSES should not supply robot-specific effect estimates without a clearly reported subgroup (28–30).

### 5.4 Specimen protection and route-related safety

The intended wound benefit must be balanced against contamination, tumor disruption, extraction-route injury and anastomotic complications. Protective sleeves or bags, avoidance of direct tumor contact and excessive compression, and assessment of specimen integrity are central technical safeguards. Adequate margins and the intended nodal clearance take priority over completing natural-orifice retrieval. These measures are procedural principles; their use does not by itself demonstrate the absence of peritoneal or local recurrence risk (7, 17).

Future reporting should distinguish planned from completed NOSES, describe conversions to abdominal extraction, and document extraction route, specimen dimensions, intraoperative contamination and route-specific functional outcomes. Reliable oncological assessment requires adequate follow-up for local, pelvic and peritoneal recurrence, not simply negative margins at discharge. Sexual and anal function, incisional hernia and quality of life also need sufficiently long follow-up and validated measures.

## 6 Postoperative recovery and complication management

### 6.1 Pain control and enhanced recovery

Recovery reflects the entire perioperative pathway. Multimodal opioid-sparing analgesia, mobilization, appropriate fluid management and timely oral intake are components of enhanced recovery practice regardless of operative platform. Differences in these cointerventions can alter pain, bowel recovery and length of stay, and should be described in comparative studies (31).

Avoiding an extraction incision offers a plausible explanation for less pain after NOSES, supported by both the recent randomized trial and selected comparative cohorts. Nevertheless, the magnitude of benefit depends on the comparator incision, analgesic regimen and patient population. A prospective case-matched NOSES study reported favorable quality-of-life and psychological outcomes, but its comparison with conventional laparoscopy does not establish an additional benefit from robotics (18, 21, 32).

### 6.2 Recovery of postoperative bowel function

Earlier first flatus or diet resumption should be distinguished from prevention of postoperative ileus or mechanical bowel obstruction. These endpoints have different definitions and clinical implications. A retrospective colectomy analysis found a lower ileus rate with robotics in left-sided resections but not a statistically significant difference in right-sided resections. The robotic NOSES meta-analysis did not demonstrate a reduction in postoperative ileus. A general claim that robotic surgery prevents ileus or bowel obstruction is therefore unwarranted (10, 26).

Inflammatory markers are also intermediate measures. The 298-patient cohort reported by Cuk et al. associated robotic surgery with a lower postoperative C-reactive protein response, but did not show a significant difference in short-term surgical or medical complication rates. Lower inflammatory markers should not be treated as proof of reduced adhesions, improved autonomic function or fewer anastomotic leaks (33).

### 6.3 Incidence and management of postoperative complications

Complication rates should be reported by procedure, severity, denominator and follow-up interval. REAL found fewer 30-day complications of Clavien-Dindo grade II or higher with robotic rectal surgery, whereas ROLARR and COLRAR did not demonstrate a significant overall complication advantage. These findings do not justify a uniform complication-reduction claim across colon and rectal operations. Similarly, the recent robotic NOSES trial found no significant difference in overall complications or anastomotic leakage despite favorable wound outcomes (3–5, 21).

Robotic dexterity does not eliminate anastomotic failure. In a retrospective series of 636 robotic mid-low rectal resections, leakage occurred in 6.0%; male sex, lower tumor location and longer operative time were associated with greater risk. NOSES was not significantly associated with leakage in that selected cohort. These observations inform risk assessment but do not establish that extraction route or platform is causally protective (34).

Management follows the same clinical principles as after other colorectal operations. Early recognition and assessment of clinical stability, imaging findings and source control guide treatment. Selected stable patients with a contained leak may be managed with antibiotics and, when indicated, image-guided drainage. Generalized peritonitis, instability or failure of nonoperative treatment usually requires operative source control, with drainage, diversion or anastomotic revision or resection according to the findings (35). Wound problems, persistent bowel dysfunction and route-related symptoms after NOSES also require assessment. Urinary, sexual and bowel function should be followed beyond the initial admission; a technically successful operation does not establish functional preservation.

## 7 Ongoing controversies and implementation

### 7.1 When does a technical advantage become a clinical benefit

The principal controversy is outcome-specific. The positive recurrence result from REAL should be acknowledged alongside the neutral primary outcomes of ROLARR and COLRAR, rather than replaced by a blanket assertion that all robotic evidence is weak. Conversely, the REAL result should not be generalized to every rectal operation, all surgeons or newer platforms. Rectal cancer trials also do not answer whether robotics improves colectomy outcomes. Table 1 makes these distinctions explicit (3, 4, 6, 9).

Future trials should define a clinically meaningful primary endpoint and record surgeon experience, operative technique and perioperative care. A small difference in blood loss or time to first flatus may not translate into fewer major complications, better function or lower resource use. Patient-reported outcomes and recovery trajectories should be considered alongside pathological quality and cancer recurrence.

### 7.2 Does robotic assistance justify wider lymphadenectomy

Two questions must remain separate: whether a particular dissection extent benefits a defined patient group, and whether robotics improves delivery of that dissection. Guideline differences and the RELARC findings show why feasibility alone cannot resolve the first question. A robotic versus laparoscopic study should compare equivalent intended nodal territories before attributing pathological or survival differences to platform choice (1, 12, 14, 15).

For rectal cancer, lateral pelvic clearance requires its own indication and assessment of local control and autonomic function. For fluorescence-guided surgery, an altered drainage map is a finding to investigate, not automatic authorization for wider resection. The evidence reviewed does not establish that higher node counts are the mechanism underlying the oncological benefits seen in REAL.

### 7.3 Can NOSES benefits be reproduced outside selected centers

The new randomized short-term evidence is encouraging, while the longer-term evidence remains largely observational. The unresolved question is whether the balance of wound benefits, extraction-related harms and cancer outcomes remains favorable across different anatomy, body habitus, tumor burden, extraction routes and levels of experience. Studies should include the denominator of all potentially eligible patients and the reasons NOSES was not attempted or completed (20, 21, 24, 25).

An adequately powered long-term comparison should use a prespecified noninferiority framework if the aim is to establish oncological noninferiority. It should report both absolute risks and uncertainty, account for exclusions and crossover, and use consistent recurrence and functional definitions. Existing nonsignificant survival comparisons cannot substitute for that design. Overlap between institutional cohorts should also be checked before interpreting multiple reports or pooled estimates as independent evidence.

### 7.4 Costs and patient selection

Higher acquisition, maintenance and consumable costs remain relevant to implementation. In the small randomized right-colectomy study, greater robotic expenditure was not offset by a demonstrated recovery benefit. More recent administrative data in very elderly patients associated robotics with shorter hospitalization but higher total hospital costs, without significant differences in mortality or perioperative complications. Shorter stay therefore does not necessarily establish cost-effectiveness (8, 36).

Patient subgroups should not be selected solely on plausible technical benefit. A propensity-matched analysis in morbidly obese patients did not find a significant overall complication advantage for robotics and reported higher costs. Anatomy, comorbidity, available expertise and patient preferences should inform the decision, while claims of preferential benefit require direct comparative support (37). Economic evaluations should include the full care episode, operating-room use, complications, readmissions, functional recovery and platform utilization, with separate analyses for colectomy, rectal resection and NOSES.

### 7.5 Learning curves, consent and quality assurance

Learning-curve estimates depend on prior experience, access to the platform, team organization, procedure complexity and the metric used to define proficiency. A retrospective rectal series identified an operative-time plateau after 49 cases. A robotic NOSES series reported different turning points for two surgeons, at 15 and 42 cases. These values describe particular settings and should not become universal credentialing thresholds. Faster operating time alone is insufficient evidence of competence in oncological dissection or complication management (38, 39).

Implementation should combine supervised training with monitoring of conversion, specimen quality, major complications, readmission and function. Consent should explain the proposed platform and extraction route, available alternatives, expected recovery advantages and remaining uncertainty. For NOSES, this includes the possibility of abdominal extraction and route-specific functional concerns. Transparent reporting of institutional experience and outcomes is more informative than attributing safety to technological sophistication (7).

## 8 Emerging platforms and future research

### 8.1 Platform diversification and image guidance

Emerging systems may broaden access, but evidence should be interpreted at the level actually studied. A 103-patient retrospective comparison associated the Weigao system with lower hospitalization costs and longer operating time than da Vinci; it did not establish long-term oncological equivalence. A *post hoc* analysis of a 100-patient randomized Kangduo comparison found no significant difference in trajectories of systemic stress markers, which addresses a physiological endpoint rather than survival. A propensity-matched hinotori comparison likewise reported short-term findings and longer operative time, with long-term outcomes still requiring evaluation (40–42).

Single-port surgery, fluorescence integration and enhanced navigation should be evaluated against specific clinical problems. Smaller access incisions or improved visualization are not sufficient endpoints for claiming reduced leakage, recurrence or costs. Platform studies should report both technical success and adverse events, and distinguish initial adoption from established use.

### 8.2 Molecular information and future decision support

Molecular characterization contributes to systemic treatment decisions in colorectal cancer, but the guidelines reviewed do not establish a molecular-profile-based template for robotic lymphadenectomy or an indication for choosing robotics over laparoscopy. In current operative planning, anatomy, disease extent, imaging, treatment response and the intended oncological resection remain central. A proposed link between molecular subtype and the extent of robotic nodal clearance should be identified as a research hypothesis (12).

Artificial intelligence, augmented reality and navigation tools may eventually assist anatomical recognition or intraoperative decision-making. Their value in this setting requires prospective evaluation against conventional planning and visualization, with attention to accuracy, failure modes and clinically meaningful outcomes. Such concepts should be kept distinct from established surgical practice and should not expand the scope of lymphadenectomy without supporting evidence.

## 9 Conclusion

Robot-assisted surgery provides useful technical capabilities, with the strongest comparative evidence concentrated in rectal cancer. REAL supports selected perioperative and 3-year oncological benefits in middle and low rectal cancer, while other randomized trials and colectomy studies limit claims of universal superiority. Lymph node yield, vascular ligation, anatomical dissection extent and long-term cancer control must be assessed separately. Current evidence does not establish that wider or higher-yield robotic lymphadenectomy itself improves survival.

Robotic NOSES can reduce the abdominal extraction burden in selected patients. Recent randomized findings support short-term recovery and wound benefits, but long-term oncological noninferiority and wider generalizability remain unresolved. Appropriate resection, specimen protection, patient selection and institutional capability should determine its use. Further research should isolate the contributions of platform, dissection strategy and extraction route, and prioritize recurrence, function, major complications and cost-effectiveness.
